# Thymic B cell development is controlled by the B potential of progenitors via both hematopoietic-intrinsic and thymic microenvironment-intrinsic regulatory mechanisms

**DOI:** 10.1371/journal.pone.0193189

**Published:** 2018-02-20

**Authors:** Shiyun Xiao, Wen Zhang, Nancy R. Manley

**Affiliations:** Department of Genetics, Paul D. Coverdell Center, University of Georgia, Athens, Georgia, United States of America; RIKEN Center for Integrative Medical Science, JAPAN

## Abstract

**Background:**

Hematopoietic stem cells (HSCs) derived from birth through adult possess differing differentiation potential for T or B cell fate in the thymus; neonatal bone marrow (BM) cells also have a higher potential for B cell production in BM compared to adult HSCs. We hypothesized that this hematopoietic-intrinsic B potential might also regulate B cell development in the thymus during ontogeny.

**Methods:**

*Foxn1*^*lacZ*^ mutant mice are a model in which down regulation of a thymic epithelial cell (TEC) specific transcription factor beginning one week postnatal causes a dramatic reduction of thymocytes production. In this study, we found that while T cells were decreased, the frequency of thymic B cells was greatly increased in these mutants in the perinatal period. We used this model to characterize the mechanisms in the thymus controlling B cell development.

**Results:**

*Foxn1*^*lacZ*^ mutants, T cell committed intrathymic progenitors (DN1a,b) were progressively reduced beginning one week after birth, while thymic B cells peaked at 3–4 weeks with pre-B-II progenitor phenotype, and originated in the thymus. Heterochronic chimeras showed that the capacity for thymic B cell production was due to a combination of higher B potential of neonatal HSCs, combined with a thymic microenvironment deficiency including reduction of DL4 and increase of IL-7 that promoted B cell fate.

**Conclusion:**

Our findings indicate that the capacity and time course for thymic B-cell production are primarily controlled by the hematopoietic-intrinsic potential for B cells themselves during ontogeny, but that signals from TECs microenvironment also influence the frequency and differentiation potential of B cell development in the thymus.

## Introduction

The thymus is the primary site of T cell development, differentiation, and maturation, and is seeded periodically by lymphoid progenitor cells (LPCs) from outside the thymus [1–4]. At least three discrete waves of LPCs seed the thymus at different stages from various hematopoietic tissues including the Aorta-gonado-mesonephros region (AGM), fetal liver (FL), and bone marrow (BM) [5,6], each of which has distinct lineage potentials [7–9]. A developmental switch from fetal to adult HSCs occurs during the first to three weeks of postnatal life in mice [10–12]. Adult HSCs differ from fetal HSCs in number and phenotype, and thymus-seeding LPCs derived from adult HSCs possess multiple lineage potentials for the development of T/B/NK/DC and myeloid cells within the thymus [13–16]. HSCs demonstrate an age-related decrease in B lineage potential between neonatal BM or cord blood and adult BM [7,17,18]. Fetal HSCs also preferentially develop into B-1a type B cells, rather than the more conventional postnatal B-2 (referred to as B) cells [19,20]. Thymic seeding progenitors (TSPs) in the neonatal thymus also appear to have higher B potential than those from adult thymus [21,22]. However, how does TSPs in variable potential undergo the B lineage commitment and expansion, and be regulated by the thymic environment during neonatal to young adult is still unclear.

The vast majority of LPCs commit to a T cell fate upon entering the thymus via activation of the Notch signaling pathway. Notch signaling between LPCs expressing Notch receptors and thymic epithelial cells (TECs) expressing the Delta-like 4 (DL4) ligand is required for LPCs to commit to the T lineage [23–25]. In the absence of Notch signaling, LPCs undergo B lineage commitment in the thymus. TEC differentiation, proliferation, and functional maintenance are dependent on TEC-specific transcription factor FOXN1 [26]. *Foxn1* down-regulation at either fetal or postnatal stage reduces *Dl4* expression, which leads to an increase in thymic B cells [27–29], specifically B-1a cells [27]. In addition to the direct loss of Notch signaling, overexpression of IL-7, TCRβ deficiency, and CD3ε mutants have all been shown to promote B cell development in the thymus [30–32]. The wild-type adult thymus also produces a small number of B cells (<1% of total thymocytes, ~2 x 10^4^ per day) that are exported to the periphery [31]. Thymic B cells normally reside preferentially at the cortical-medullary junction and express a high level of MHCII. Although their functional role in the thymus is not entirely clear, thymic B cells have been recently implicated in negative selection during T cell development [33–35]. However, the mechanisms that normally regulate B cell development in the thymus, and the role of TECs in this process, are not known.

We previously generated a novel *Foxn1* allele, designated *Foxn1*^*lacZ*^ [36]. In this model, *Foxn1* expression is normal at fetal stages, but down-regulated beginning postnatal day 7, causing progressive involution of the thymus and reduction of total thymocytes. In the current study, we show that the earliest TCRαβ committed progenitors (DN1a/b [16]) generate a wave of T cells around day 7 in both controls and *Foxn1*^*lacZ*^ mutants accompanied by a transient increase in thymic B cells. In the current study, we addressed the developmental kinetics and the capability of production of thymic B cells, characterized this abnormal increase in B cells, and identified their origin and the regulatory molecules in the thymus. We provide evidence that although DL4 and IL-7 levels influence the production of thymic B cells, the intrinsic B potential of HSCs during neonatal versus young adult ages is key for the development of thymic B cells. Our data demonstrate that the production of thymic B cells is controlled not only by changes in the B lineage potential of HSCs cells, but also by factors expressed by the thymic epithelium that regulate B cell fate and developmental progression during the transition from neonatal to young adult.

## Material and methods

### Mice

*Foxn1*^*lacZ/lacZ*^ (Z/Z) mice were generated as described previously on a C57Bl6/J background [36,37]. *Foxn1*^*nude*^ heterozygous (*Foxn1*^*+/nude*^*)* mice on a C57Bl6/J background (Stock No: 000664) were purchased from The Jackson Laboratory (Bar Harbor, ME). *Foxn1*^*lacZ/nude*^ (Z/N) mice were generated by crossing Z/Z with *Foxn1*^*+/nude*^ mice. CD45.1 C57BL6/J mice (Stock No: 002014) were purchased from Jackson Laboratory (Bar Harbor, ME). All analysis was performed on littermate animals whenever possible. All mice were maintained in a specific pathogen-free (SPF) facility at University of Georgia. All experiments were approved by the University of Georgia Institutional Animal Care and Use Committee.

### Flow cytometry

Freshly isolated thymocytes in suspension (1×10^6^) were used for each sample. Cells were blocked by anti-CD16/32 (Clone:93) antibody before staining. For tracing the kinetic phenotypic profile and counting the numbers of thymocyte subsets, anti-CD4 APC (GK1.5), anti-CD8 FITC (53–6.7), anti-CD44 PE (IM7), and anti-CD25 biotin (3C7) followed by streptavidin PerCP were used. For analyzing the profile of Lin^-^ DN1ab T cells in the total DN1subpopulations, phycoerythrin (PE) conjugated lineage markers anti-CD3 (145-2C11), CD4, CD8, CD11c (N418), CD19 (6D5), Gr-1 (RB68-C5), TER-119 (TER-119), NK1.1 (PK136) plus anti-CD25 PE antibodies were mixed and combined with anti-CD44 biotin followed with streptavidin PerCP. The profile of DN1a through DN1e subsets was analyzed using anti-cKit-APC (2B8) and CD24-FITC (M1/69) antibodies. For analysis of thymic B cells in total DN1 cells, PerCP conjugated anti-CD4, CD8 and CD25 antibodies with anti-CD19 PE, or NK1.1 PE and CD44-APC, CD24-FITC were used. Total thymic cells gated on CD19^+^B220^+^ (RA3-6B2) were also analyzed for CD43 (1B11), CD93 (AA4.1), Ly51 (6C3), CD25, MHCII (M5/114.15.2), IgM (RMM-1), IgD (11-26c.2a) or CD5 (53–7.3) in a different panel. All antibodies were purchased from Biolegend (San Diego, CA). The data were analyzed using CellQuest^TM^ or Flowjo^TM^ software.

### Cell sorting

For thymic B-cell sorting, a total thymus suspension was passed through a cell strainer, and cells were stained by CD19 PE-Cy7, B220 APC, IgM + Lin PE, CD24 FITC and CD43 APC-Cy7 (1B11). Thymic progenitor B cells were gated on CD19^+^B220^+^CD24^+^CD43^+/lo^IgM^-^ subpopulations. For LSK cell transfer, BM suspension cells from 14 day and 3 months Ly5.1 mice were isolated and stained with PE-conjugated antibodies for the lineage markers cKit-APC and Sca-1-FITC (D7, Biolegend). The LSK cells were gated on the Lin^-^Sca1^+^cKit^+^ subpopulation. All cells were sorted using a MoFlo^TM^ cell sorter.

### Chimera generation

BM cells were isolated from CD45.1 mice at indicated ages, and RBCs deleted by ACK-lysing buffer (Cambrex Bio Science). To isolate T and B deleted BM cells, the suspension of BM cells was incubated with purified rat anti-CD19 and CD3 antibodies and followed by anti-rat IgG Dyna-beads (Invitrogen Life Technologies). The efficiency of T/B cell removal was higher than 95%. 1×10^7^ T/ B-depleted BM cells in 200μl PBS from CD45.1 mice at a different age were retro-orbital transferred into sub-lethal (600 rad) irradiated CD45.2 mice at indicated age. Donor (CD45.1) and host thymic cells (CD45.2) were analyzed 12 days after cell transfer. For LSKs transfer, sorted 14-day or 3-month CD45.1 LSK cells were transferred respectively into sub-lethally irradiated 1.5 month or 17-day old Z/Z mice by retro-orbital injection, 3000 LSK cells per mouse. The donor and host thymic B cells were analyzed 6 weeks after transfer. For competition experiments, BM cells from 3 months CD45.1 and day 20 CD45.2 BL6 were mixed and then transferred into lethally (1100 Rad) irradiated Z/Z mice, then analyzed 12 days after transfer.

To identify if the increased thymic progenitor B cells in mutants were seeded by circulating progenitor B cells from BM or spleen, The B220^+^CD19^+^CD24^+^CD43^+/lo^ IgM^-^ progenitor B cells were sorted from CD45.1 BM and spleen. 5 × 10^6^ mixed pre-B cells were then retro-orbital injected into sub-lethal irradiated (600 Rad) adult Z/N (CD45.2) mice. 21 days later, the donor cells were analyzed in the host.

### Immunofluorescence

Thymi were removed and placed in OCT, frozen on dry ice, then sectioned at 10μm. Primary antibodies rat anti-CD19, rabbit anti-K5 (AF138, Covance) or rabbit anti-β5t (polyclone, MBL) followed by appropriate fluorescence-conjugated donkey secondary antibodies (Jackson Immunoresearch) were used. Images were obtained using a Zeiss Axioplan2 imaging microscope with an AxioCam HRM, and AxioVision Rel 4.5 software (Jena, Germany).

### Statistical analysis

All data were collected in a Microsoft Excel file and analyzed using Prism software by one-way analysis of variance (ANOVA)-Bonferroni test or student’s t-test, P value in two-Tailed.

## Results

### Foxn1 down-regulation results in a transient increase in DN1 thymocytes

**Fig 1:**
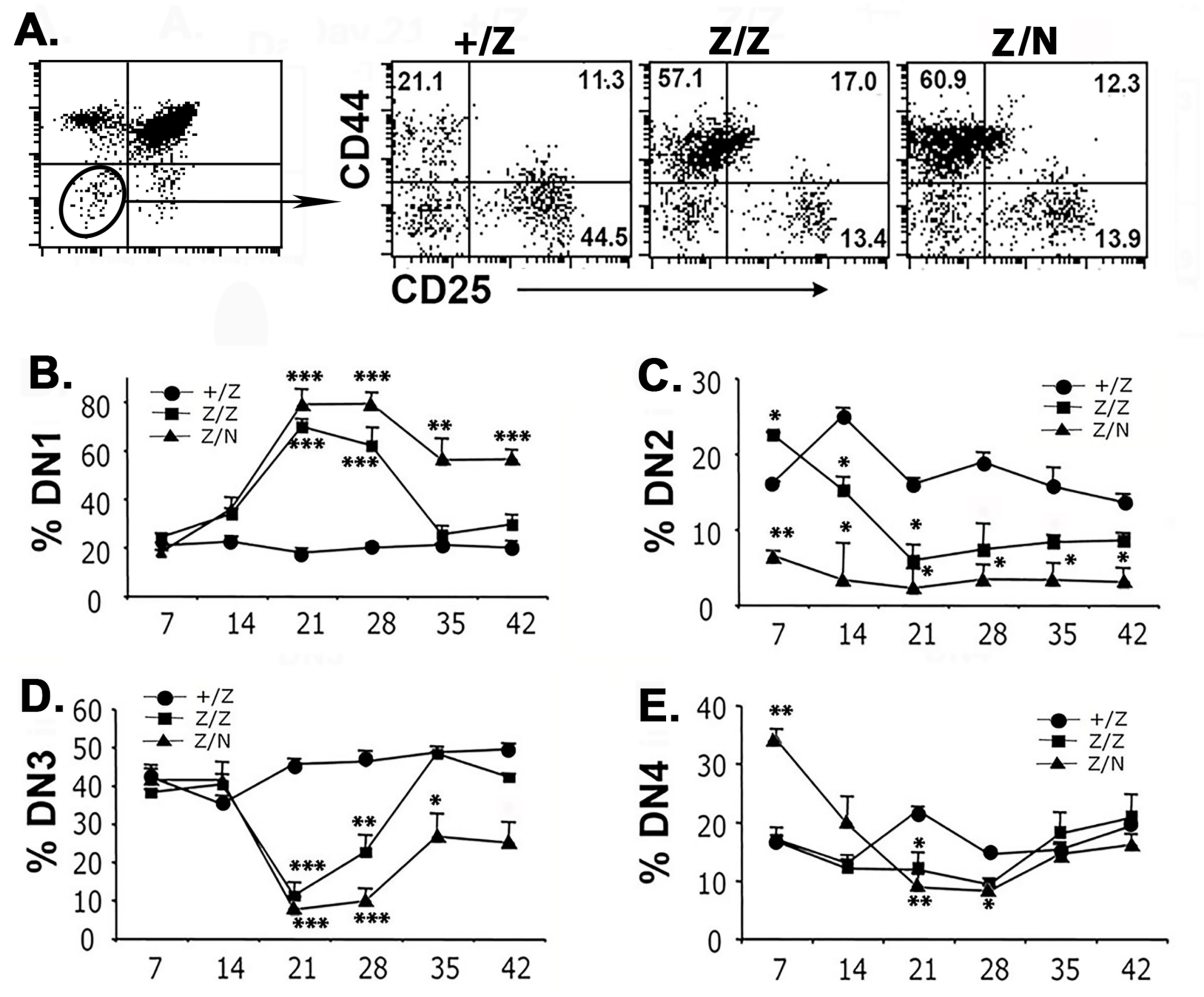
Transient increase of DN1 and decrease of DN2, DN3 showed in *the Foxn1*^*lacZ*^ thymus. **(A).** Representative profiles of CD25 and CD44 on gated total CD4^-^CD8^-^ DN thymocytes at day 21 in +/Z, Z/Z and Z/N mice. **(B-E).** Percentages of DN1, DN2, DN3, and DN4 populations at various time points are summarized in the histogram. Each assay and time point represents at least 5 individuals. One-way ANOVA results between Z/Z, Z/N test groups and +/Z control group at various time point: *P <0.05, **P <0.01, ***P <0.001. Bars indicate means ± SEM.

The *Foxn1*^*lacZ*^ allele causes a gradual down-regulation of *Foxn1* expression in TECs beginning one week after birth, resulting in a rapid reduction of total thymocyte numbers, but a transient increased frequency of CD4^-^CD8^-^ DN cells that peaks at 21–28 days [36]. To investigate this increase in more detail, we analyzed the DN subsets during this time frame in both *Foxn1*^*lacZ/lacZ*^ (Z/Z) and *Foxn1* ^*nude/lacZ*^ (Z/N) mutants, which have one copy of a null allele and so have further reduced *Foxn1* levels, and compared them to *Foxn1*^*+/lacZ*^ (+/Z) control mice. This transient increase was specifically due to an increased percentage of DN1 (CD44^+^CD25^-/lo^) cells in both Z/Z and Z/N mutants after day 7 (Fig 1A and 1B), reaching a peak of 60–80% of total DN cells at day 21–28. However, DN1 and DN3 percentages were almost restored to the control level at 5 weeks of age in Z/Z, and were partially restored in Z/N mice (Fig 1B and 1D). Interestingly, although the thymic microenvironment deficiency is more severe in Z/N mice [36], the kinetics of the transient DN1 increase and of the DN3 reduction were similar in both Z/Z and Z/N mice, indicating that the thymic microenvironment is not the cause of the kinetic changes in DN subsets.

### Thymic progenitors have reduced T but increased B cell potential after day 7 postnatal in Foxn1^lacZ^ thymus

**Fig 2:**
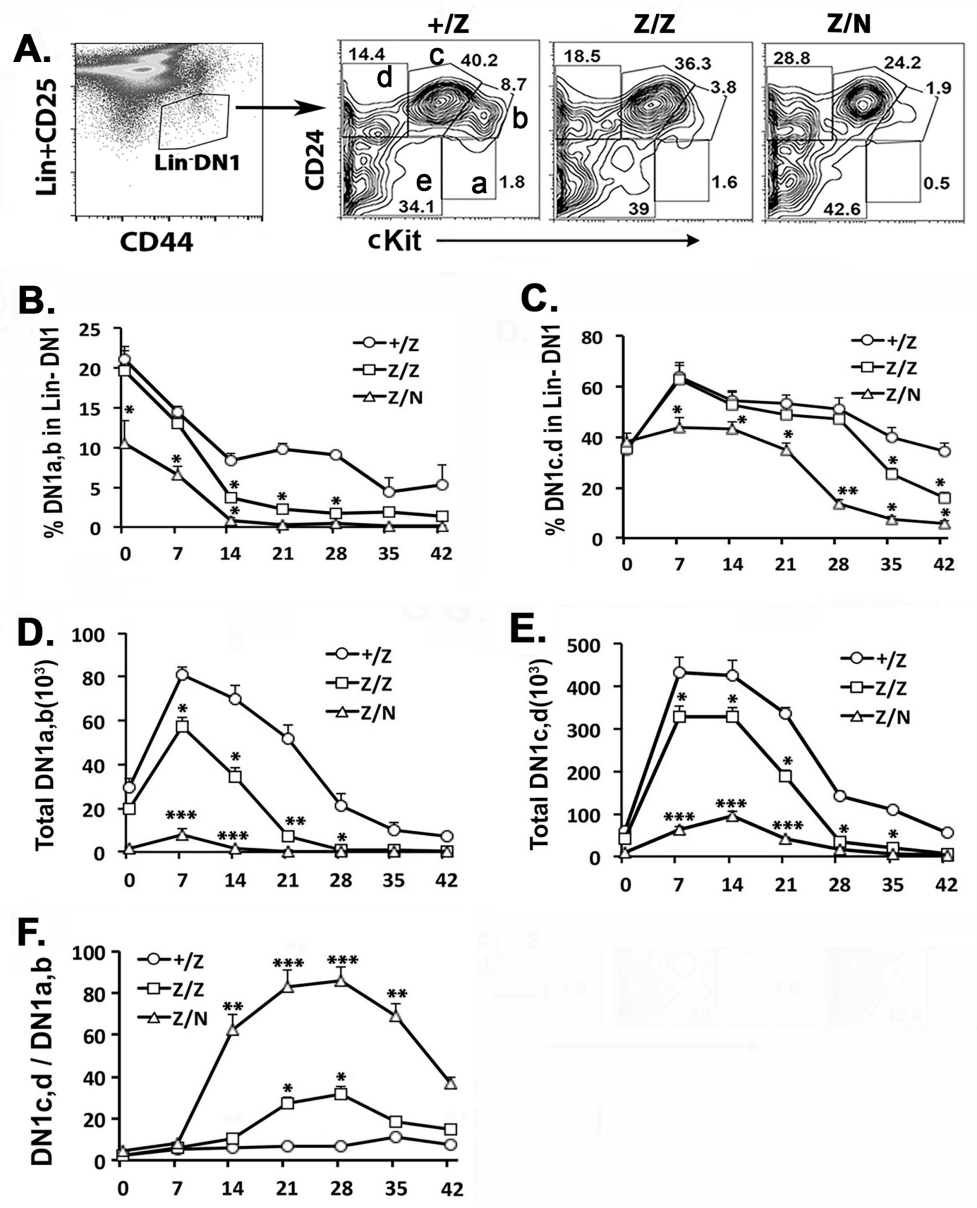
The kinetic change of DN1a,b, and DN1c,d subsets showed an increased B potential in the thymic progenitors in *Foxn1*^*lacZ*^ mutants. **(A).** The representative profiles of CD24 and cKit on gated Lin^-^DN1 population at day 14 in +/Z, Z/Z, and Z/N mice. DN1a-e subsets gates in +/Z. **(B, C).** Percentage of DN1a,b (DN1a plus DN1b) (B) and DN1c,d (DN1c plus DN1d) (C) at various analysis time points. **(D, E).** Total cell number of DN1a,b (D) and DN1c,d (E) at various analysis time points. **(F).** The ratio of DN1c,d versus DN1a,b cells. Each assay and time point represented at least three individuals. One-way ANOVA results between Z/Z, Z/N test groups and +/Z control group at various time point: *P <0.05, **P <0.01, ***P <0.001. Bars indicate means ± SEM.

The transient increase in DN1 and reduction in DN2 cells could have been due to a partial block at the DN1-DN2 transition [36]; alternatively, it could also reflect a reduction in early thymic progenitors (ETPs). cKit^hi^ DN1a,b subsets represent ETPs that commit to the TCRαβ T cell lineage and are the precursors of DN2 cells as well as most thymic NK cells, while DN1c,d cells primarily have B cell potential in DN1 [16]. By gating on Lin^-^ DN1 cells (Fig 2A), we showed that in all genotypes, the percentage of DN1a/b cells was highest at the newborn stage, and their frequency declined faster in Z/Z mice between 7–14 days, consistent with the timing of *Foxn1* expression reduction in these mice (Fig 2B). The percentage of DN1c,d cells in +/Z and Z/Z mice peaked slightly later, at day 7 (Fig 2C). However, the absolute cell number of both DN1a,b and DN1c,d subsets peaked at day 7 in both genotypes. These numbers were significantly reduced in the *Z/Z* mutants (Fig 2D and 2E). Furthermore, the rate of decline in total numbers was slower in DN1c,d cells than in DN1a,b cells in Z/Z mutants from day 14 through 21, resulting in an increased ratio of DN1c,d to DN1a,b in Z/Z after day 7 (Fig 2F). These effects tended to be exacerbated by further declines in the microenvironment in Z/N mice. The peak timing of DN1a,b and DN1c,d subset frequency was similar to +/Z and Z/Z mice (Fig 2B and 2C), although the frequency and number of DN1a,b cells was strongly reduced in these mice (Fig 2B and 2D). The timing of the peak of DN1c,d numbers was also shifted later, to 14 days (Fig 2E). The net effect of these differences was that the DN1c,d/DN1a,b ratio was dramatically increased beginning at day 14 (Fig 2F)

These data suggest that even in the controls, the frequency of cells biased to the B cell lineage peaks at 7–14 days postnatal. Furthermore, the degree of B cell bias is increased in the Z/Z and Z/N mutants with defects in the microenvironment, with timing that corresponds to the down-regulation of *Foxn1* in these mutants [36]. Taken together, these data implicate both cell-autonomous and non-autonomous mechanisms may influence thymic B cell production during this perinatal period.

### A transient increase in thymic B-cell production during the neonatal to young adult transition

**Fig 3:**
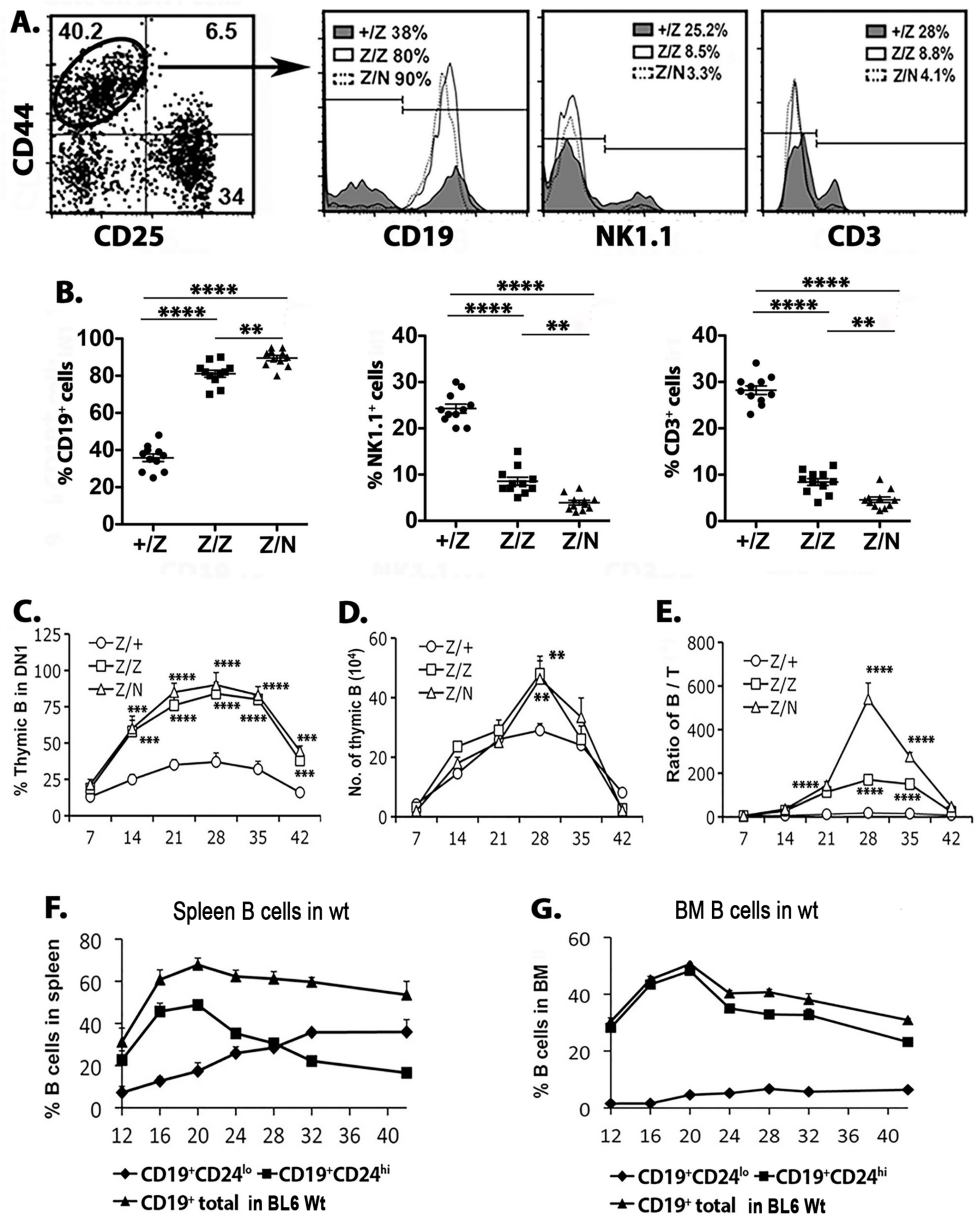
Thymic B cells formed a transient wave around day 28 in the thymus. **(A).** Histograms show the representative staining of CD19, NK1.1 and CD3 on gated DN1 thymocytes in +/Z, Z/Z and Z/N mice. **(B).** The summary of the percentage of CD19^+^, NK1.1^+^ and CD3^+^ cells in the DN1 subset. **(C-E).** Kinetic change of the percentage of thymic B cells in DN1 subset cell (C), total thymic B-cell number (D), and the ratio of thymic B cells to total thymocyte number (E) in +/Z, Z/Z, and Z/N mice. Each assay time point represents at least five individuals. **(F-G).** The kinetic change of the percentage of total B cells (CD19^+^), mature B cells (CD19^+^CD24^lo^) and immature B cells (CD19^+^CD24^hi^) in spleen (F) and BM (G) cells in BL6 mice. Each assay time point represents at least three individuals. One-way ANOVA results between Z/Z or Z/N test group and +/Z control group at various time point: *P <0.05, **P <0.01, ***P <0.001, ****P<0.0001. Bars indicate means ± SEM.

Based on the analysis above, we directly assessed B cell development in the thymus during this period and in *Foxn1*^*lacz*^ mutants. Analysis of total DN1 cells showed that at day 28 more than 80–90% of DN1 cells were CD19^+^ in *Foxn1*^*lacz*^ mutant mice, compared to 38% in the +/Z controls, and that NK and CD3^+^ cells were relatively reduced in both Z/Z and Z/N mutants (Fig 3A and 3B). These results confirmed that increased B cells were the primary cause for the increase in the DN1 subset during this window. Further analysis of the kinetics of thymic B-cell production showed that the percentage of B cells started increasing one week after birth and reached a peak within DN1 cells at day 28 in all genotypes analyzed (Fig 3C). Although the total thymic cell number was much less in *Foxn1*^*lacZ*^ mutants than in the control thymus [36], the numbers of thymic B cells were almost 2-fold higher in both Z/Z and Z/N mutants at day 28 (Fig 3D), resulting in a much higher ratio of thymic B cells to T cells than in +/Z control thymus, particularly in the Z/N mice (Fig 3E). However, the peak time of thymic B-cell production was similar in all genotypes, at 28 days. These results strongly suggested that changes in the thymic environment in these mutants controlled the magnitude, but not the timing of thymic B-cell production.

It has been reported that the highest absolute numbers of clonable progenitor B cells in the BM is around 3–4 weeks of age [17]. To determine if the production kinetics of thymic B cells correlated with the ontogeny of B cell development in the BM, we compared the kinetics of thymic B cells to the development of B cells in the spleen and BM (Fig 3F and 3G). Newly produced CD19^+^CD24^hi^ immature B cells increased from day 12 with a peak at day 20 in both the spleen and BM. Thus, B cell production in both the spleen and BM preceded that in the thymus, likely due to the time needed for BM progenitors to seed the thymus. B cell development in the thymus may also be slower than in BM. Since the importation of BM-derived progenitors in the postnatal thymus is a gated phenomenon, profiled as 7 days of receptivity with around 4 weeks of refractivity [4,6], the wave of thymic B cells around day 28 was likely generated from neonatal BM-derived progenitors.

### The progenitors from young BM possess higher potential to generate B cells in the thymus

**Fig 4:**
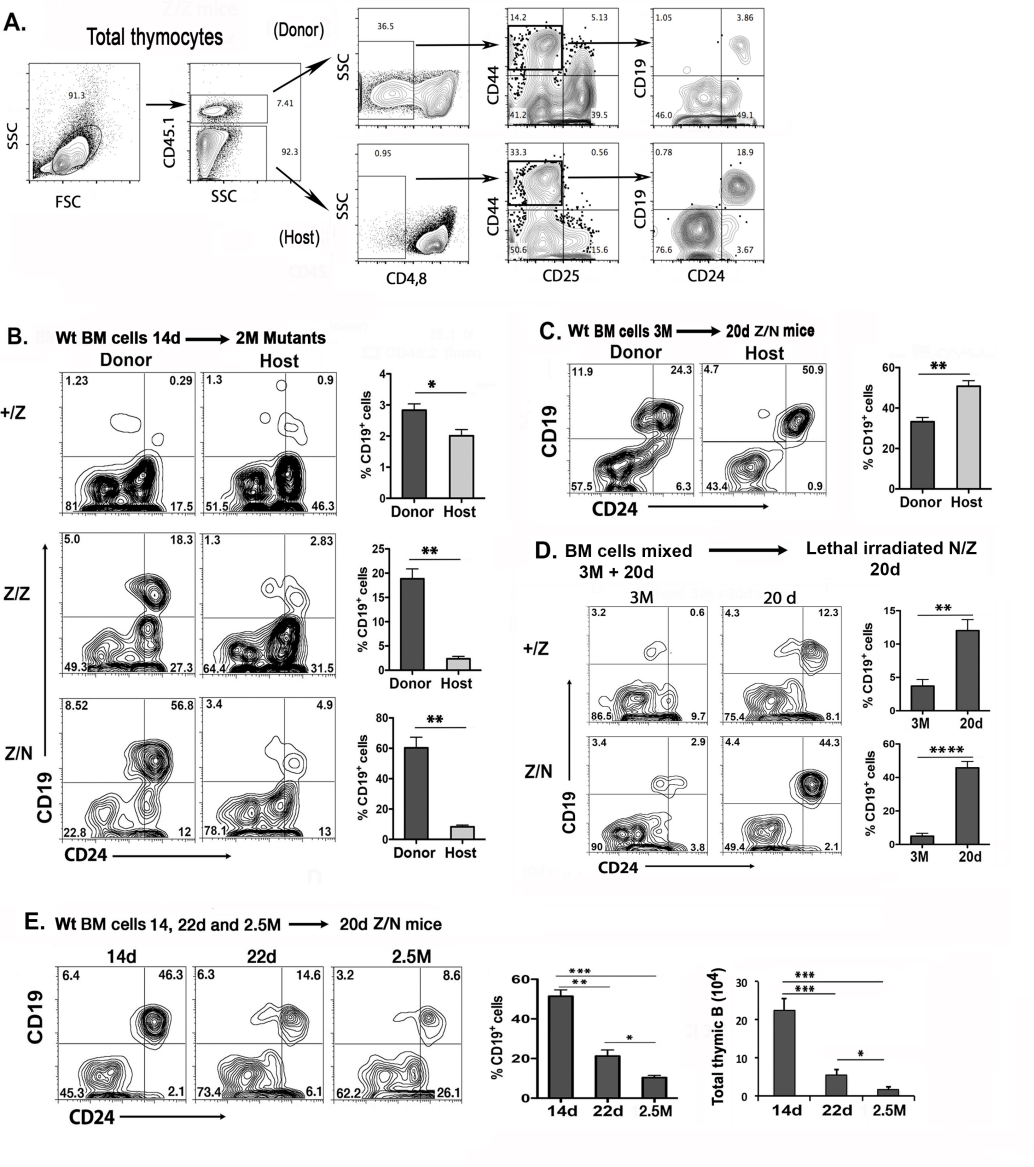
Young BM cells generated more thymic B cells than adult BM cells due to an age-dependent B potential revealed by the *Foxn1*^*lacZ*^ thymus. 1x10^7^ T and B-depleted BM cells from age indicated CD45.1 mice were transferred into sub-irradiated CD45.2 +/Z, Z/Z, and Z/N mice. Thymic B cells from the donor (CD45.1) and host (CD45.2) were analyzed 12 days later. **(A).** Gating for donor and host CD19^+^ cells in the DN1 subset. **(B).** Day 14 wild-type BM cells were transferred into 2-month old mice, genotypes of host recipients are indicated to the left of each pair of panels. Profiles of CD19 and CD24 staining within the DN1 subset are shown. Percentages of CD19^+^ cells in DN1 subsets in donor and host are summarized in the graphs to the right. Data are representative of three independent experiments, (+/Z: n = 7, Z/Z: n = 5, Z/N: n = 4). **(C).** 3 month-old wild-type BM cells were transferred into 20-day old Z/N mice. Data are representative of two independent experiments, (n = 4). **(D).** Competitive transfer experiment of mixed BM cells. 5 x 10^6^ BM cells from 3 months CD45.1mice and 5 x 10^6^ BM cells from day 20 CD45.2 mice were mixed and then transferred into day 20 lethal irradiated Z/N (n = 4) mice. The CD19 and CD24 staining profile were measured on the DN1 subset 12 days later. **(E).** BM cells from 14, 22-day and 2.5-month were transferred into sublethally-irradiated 20-day old Z/N mice, and profiled for CD19 and CD24 staining. Percentage of donor CD19^+^ cells in DN1 subsets and total thymic B cells are shown in the bar graphs. Data are representative of four independent experiments, (14d n = 5, 22d n = 3, 2.5M n = 4). Student’s t-test results: *P <0.05, **P <0.01, ***P <0.001. Bars indicate means ± SEM.

To test whether there was a BM-intrinsic component to the transient increase in B cells in these mutants, we transferred T, B-cell depleted day 14 BM cells from CD45.1 wild-type mice into 2-month-old sub-lethally irradiated CD45.2 +/Z, Z/Z, or Z/N mice, and compared the percentages of thymic B cells from both CD45.1 donor and CD45.2 host BM cells after 12 days (Fig 4A showed a gate after transfer). The 14-day donor BM cells generated significantly more B cells than did the 2-month host BM cells in both the Z/Z and Z/N mutant thymus. In Z/N mice, 60% of DN1 cells were CD19^+^, 90% of which were CD24^hi^ immature B cells (Fig 4B). Conversely, BM cells from 3-month old mice transferred into day 20 Z/N mice generated significantly fewer B cells than the younger host BM in the *Foxn1*^*lacZ*^ thymus (Fig 4C). Similar results were obtained from transferring LSK cells (Lin^-^Sca-1^+^ckit^hi^) (S1 Fig). We also performed a direct competition between host and donor cells, mixing BM cells from 3 month-old (CD45.1) and 20 day-olds (CD45.2) wild-type mice and transferring this mixture into Ly5.2 Z/N lethally irradiated hosts, which showed similar results (Fig 4D). To determine if B cell potential in BM cells was age-dependent, T, B-depleted BM cells from 14 day, 22 day and 2.5 month-old CD45.1 wild-type donors were transferred into sub-lethally irradiated day 20 Z/N mutant host mice (Fig 4E). The greatest number of thymic B cells was derived from day 14 BM, with B cell generation declining with BM age. Young BM in this model again generated significantly more B cells in the mutant thymic microenvironment. Taken together, these results indicated that perinatal and young BM-derived cells have a high intrinsic B potential, which is revealed by the *Foxn1*^*lacz*^ mutant thymic microenvironment.

### The increased thymic B cells in Foxn1^lacz^ mutants are pre-B-II cells

**Fig 5:**
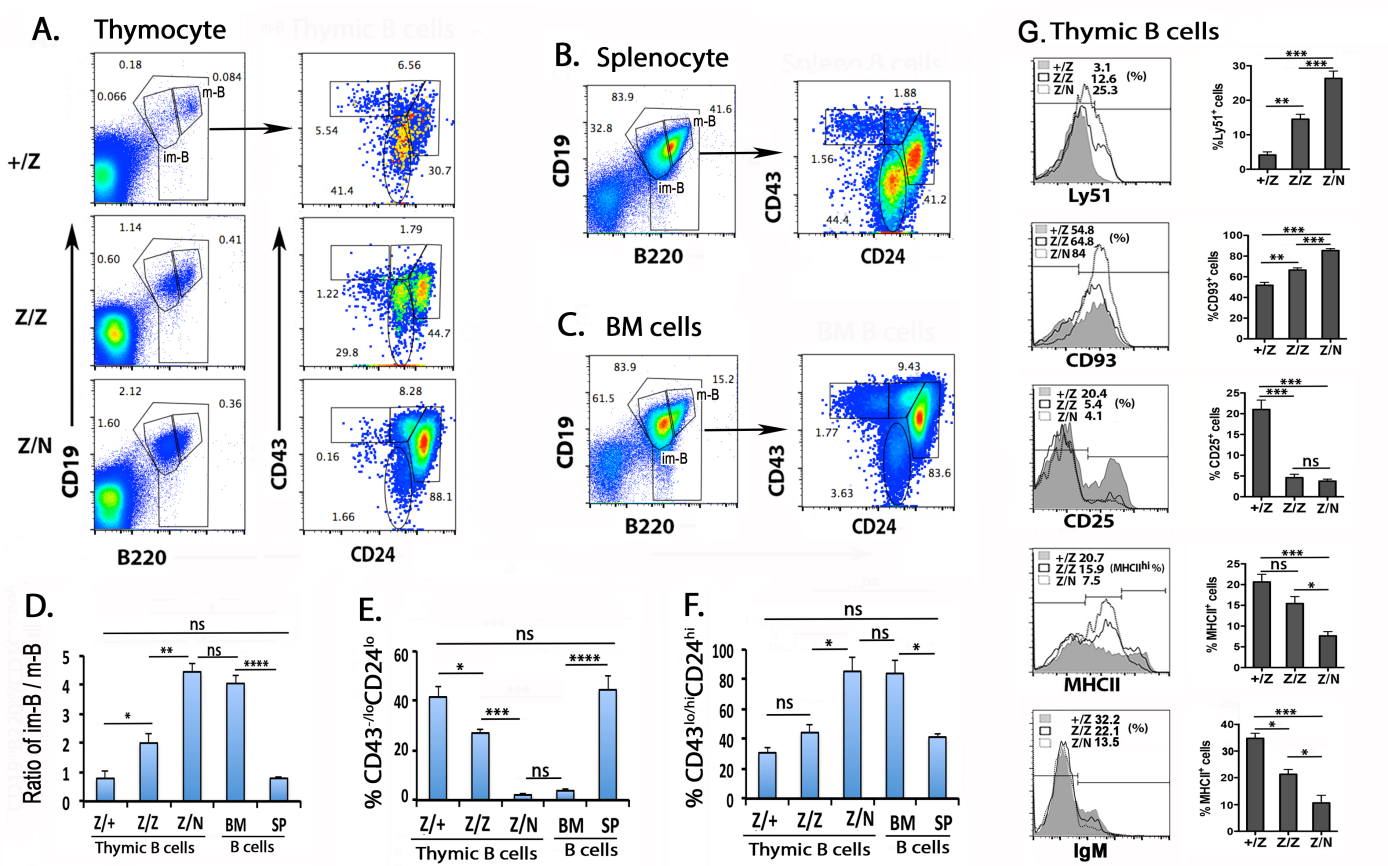
Most thymic B cells in *the Foxn1*^*lacZ*^ thymus have an immature phenotype. **(A).** Representative profiles of CD19 and B220 staining (left panels) and CD24 and CD43 (right panels) on gated B cells in thymocytes from +/Z, Z/Z, and Z/N mice as indicated to the left. **(B,C)** Same analysis of splenocytes (B) and BM cells (C) from +/Z mice. **(D).** The ratio of CD19^lo^B220^lo^ immature B (im-B) to CD19^hi^B220^hi^ mature B (m-B) cells in thymus, spleen and BM cells. **(E-F).** The percentage of CD43^-/lo^CD24^lo^ (E) and CD43^lo/hi^CD24^hi^ (F) B subsets in total thymic B cells. **(G).** Histograms show overlapping profiles of Ly51, CD93, CD25, MHCII or IgM staining on gated thymic B cells from +/Z, Z/Z and Z/N mice. All assays were performed at day 30. Data are representative of five individual experiments, (+/Z: n = 7, Z/Z: n = 5, Z/N: n = 8). One-way ANOVA: *P <0.05, **P <0.01, ***P <0.001, ****P<0.0001. ns: not significant. Bars indicate means ± SEM.

We compared the phenotypes of B cells in the thymus (Fig 5A) to the same cell population in spleen (Fig 5B) and BM (Fig 5C). No significant difference in B cell profiles were seen between +/Z controls and *Foxn1*^*lacz*^ mutants in spleen or BM; therefore, only the +/Z results in BM and spleen are shown. Most B cells in the +/Z control thymus showed a mature profile of CD19^hi^B220^hi^, and CD24 and CD43 low (Fig 5A top panel), similar to those in spleen (Fig 5B and 5D–5F). In contrast, most B cells in the Z/N mutant thymus showed an immature CD19^lo^B220^lo^CD24^hi^CD43^lo^ phenotype (Fig 5A lower panel), similar to B cells in BM (Fig 5C and 5D–5F). The phenotype of B cells in Z/Z mutants was intermediate between +/Z and Z/N (Fig 5A middle panel and Fig 5D–5F). In addition, thymic B cells in the *Foxn1*^*lacz*^ mutants had increased Ly51 and CD93 levels, but reduced IgM, CD25, and MHCII (Fig 5G). Thus, the majority of thymic B cells in Z/Z and Z/N mutants were CD19^lo^B220^lo^CD24^hi^CD93^+^CD43^lo/hi^IgM^-^, with increased Ly51, and thus are similar to the pre-B-II cells at the Fr C-D stage in BM [38–40]. However different from Pre-B-II in BM, CD25 levels were low, indicating that the development of B cells in thymus might be different from that of B cells in BM [38].

### Thymic progenitor B cells accumulate in the cortex and originate in the Foxn1^lacz^ mutant thymus

**Fig 6:**
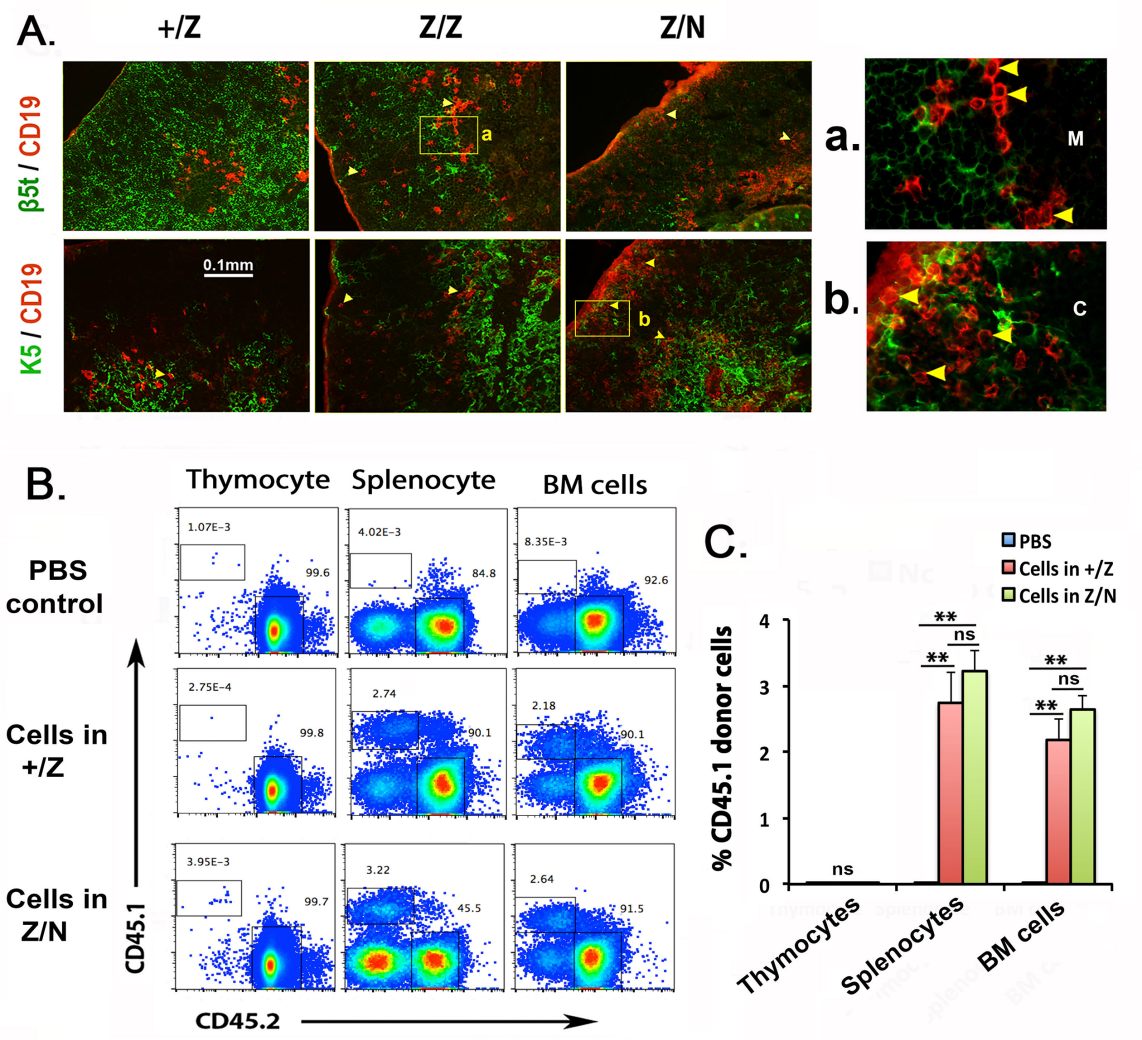
Thymic progenitor B cells accumulate in the cortex and originate in the Foxn1^lacz^ mutant thymus. **(A).** Immunofluorescence staining of sections from +/Z, Z/Z and Z/N thymus at day 30 for CD19 (red) and β5t (green) (top row), or CD19 (red) and K5 (green) (bottom row). **a,b.** Insets show digitally enlarged images of thymic B cells in the medulla in Z/Z (a) and subcapsular zone in Z/N mice (b). Scale bar = 0.1mm. **(B).** Analysis of CD45.1^+^ wild-type donor progenitor B cells (B220^+^CD19^+^ CD24^+^CD43^+/lo^IgM^-^) in thymus, spleen, and BM at 21 days after transfer into sublethally irradiated adult CD45.2 +/Z and Z/N mice (top panels: PBS injection control; middle and bottom panels: CD45.1^+^ cells transferred into +/Z and Z/N mice). **(C).** Summary of CD45.1 cell numbers from the experiment in (B). Data are representative of two independent experiments, (PBS: n = 3, cells in +/Z: n = 2, in Z/N: n = 4). One-way ANOVA: **P <0.01, ***P <0.001. ns: not significant. Bars indicate means ± SEM.

Recently, thymic B cells were reported to act as self-antigen presenting cells for the induction of central T cell tolerance [34,35,41]. Yamano showed that these thymic B cells were peripheral mature B cells that had immigrated into the thymus, different from previous studies concluding that thymic B cells were originally generated in the thymus [25,31,34]. In our study, immunofluorescence analysis showed that CD19^+^ B cells were almost exclusively localized in the β5t^-^ and K5^+^ medulla and cortical medullary junction (CMJ) in control thymus (Fig 6A left panels), while in *Z/Z* thymi, CD19^+^ B cells were distributed throughout both the cortex and medulla (Fig 6A middle panel and a). Many CD19^+^ B cells with weak staining accumulated in the subcapsular region in the *Z/N* thymus at one month of age (Fig 6A right panel and b). This distribution profile of B cells in the Z/Z and Z/N was consistent with a previous report that thymic progenitor B cells are located in the cortex [31], suggesting that the increased thymic B cells were generated within the thymus. However, the disorganized structure of the *Foxn1*^*lacZ*^ mutant thymus might allow peripheral B cells to more easily immigrate into the thymus [36]. To test whether pre-B cells can efficiently immigrate into the thymus, we isolated phenotypically similar progenitor B cells (B220^+^CD19^+^ CD24^+^CD43^+/lo^IgM^-^,) from CD45.1 spleen and BM (S2 Fig) and injected them i.v. into sub-lethally irradiated adult *Foxn1*^*lacz*^ mutants. The donor B progenitors (CD45.1) were found in both spleen and BM, but were not detected in the thymus in +/Z and Z/N mice (Fig 6B and 6C). Thus, B committed pre-B cells isolated from BM were not able to immigrate into the mutant thymus, consistent with previous reports [31,34]. Since transferred BM precursors can generate thymic B cells in *Foxn1*^*lacZ*^ mutants, we conclude that the most likely source of the increased thymic progenitor B cells was differentiation from multipotent progenitors within the *Foxn1*^*lacz*^ thymus.

### Molecular changes in key signals from the microenvironment

**Fig 7:**
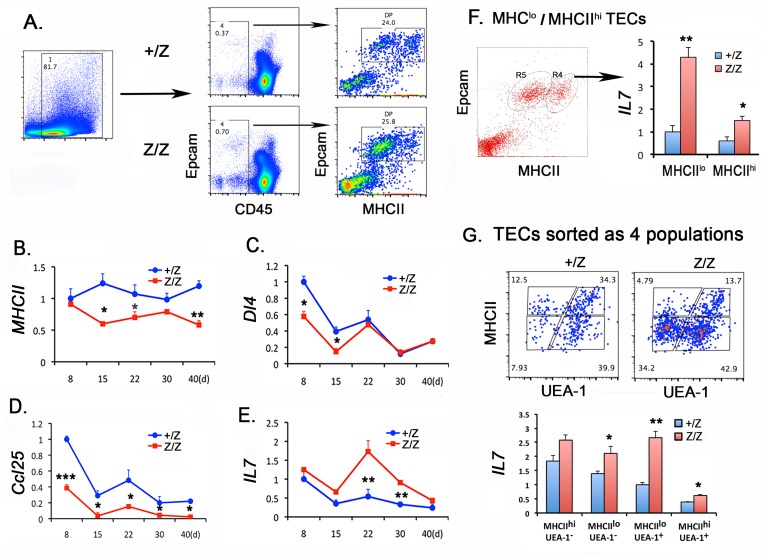
A decrease of *MHCII*, *Dl4*, and *Ccl25* but an increase in *Il7* in the *Foxn1*^*lacZ*^ thymus. **(A).** The gate for CD45^-^Epcam^+^MHCII^+^ TEC. **(B-E).** TECs sorted from day 8, 15, 22, 30 and 40 thymi were analyzed for gene expression of *MHCII (B)*, *Dl4 (C)*, *Ccl25(D)* and *Il7 (E)* respectively by qPCR. The gene expression in TECs at all time points were normalized to day 8 +/Z control values, which were set as 1. Each time point represents at least five individuals. **(F).** TECs were sorted into MHCII^lo^ and MHCII^hi^ populations from day 22 thymi. *Il7* gene expression in each sorted population is shown to the right. **(G).** TECs were sorted into four populations defined as MHCII^lo^, MHCII^hi^, cTECs (UEA-1^-^), and mTECs (UEA-1^+^) from day 30 thymi. *Il7* expression levels in these sorted populations is shown in the histogram. (+/Z: n = 2, Z/Z: n = 3 in F and G). Student’s t-test results between +/Z control and Z/Z cells: *P <0.05, **P <0.01, ***P <0.001. Bars indicate means ± SEM.

Previous studies have shown that the expression levels of factors important for LPC immigration and lineage commitment, especially MHCII, DL4, and CCL25 correlate with *Foxn1* levels ([27,28], but see [42]), and that *Dl4* and *Ccl25* are direct targets of FOXN1 [43]. To test whether the changes in T and B cell production were due to a property of the thymic microenvironment, we assessed the gene expression of above factors as well as IL-7, a key cytokine for early T and B cell expansion, in sorted total TECs (CD45^-^MHCII^+^Epcam^+^) (Fig 7A). Consistent with the timing of *Foxn1* down-regulation and reduction of MHCII^hi^ TECs in the Z/Z thymus (Fig 7A and see [36]), the *MHCII* mRNA level was reduced relative to controls after day 8 (Fig 7B). *Dl4* and *CCL25* were already lower than controls at day 8 (earlier in *Z/N* mice; data not shown), consistent with their being direct targets of FOXN1. After day 8, both *Dl4* and *Ccl25* levels dropped in both controls and mutants; while *Ccl25* levels remained lower in mutants than controls, *DL4* levels were similar by day 22 (Fig 7C and 7D). This down-regulation of *CCL25* could contribute to the reduction of ETPs in *Z/Z* mutants, while *Dl4* down-regulation at day 8 and 14 could enhance the intrinsic tendency to B cell fate in the perinatal HSCs.

Strikingly, *Il7* expression in *Z/*Z mutant TECs peaked more than 3-fold above controls at day 22 then declined, returning to control levels by day 40 (Fig 7E). Since *IL-7* is produced by TECs, especially cTECs [44–46], we sorted MHCII^lo^ and MHCII^hi^ populations from day 22 thymi to measure *Il7* expression levels. *Il7* expression was increased in both MHCII^hi^ and especially in MHCII^lo^ TECs in Z/Z mutant (Fig 7F). We further sorted MHCII^lo^ and MHCII^hi^ into cTECs (UEA-1^-^) and mTECs (UEA-1^+^) populations (Fig 7G). MHCII^lo^ cTECs and both populations of mTECs expressed significantly higher *Il7* levels in the Z/Z mutant (Fig 7G). Since MHCII^lo^ TECs are increasing in their frequency over time during in Z/Z mutants, this likely caused the increase of IL-7 in Z/Z thymus during day 14 to day 30, although the reason for the decline to baseline by day 40 is less clear.

These results suggest that the down-regulation of *Foxn1* gene expression and disorganization of the thymic microenvironment caused a broad TEC functional defect that affected ETP seeding and the commitment to B cell fate in the thymus, with a transient up-regulation of IL-7 expression that promoted immature B cell expansion in the *Foxn1*^*lacz*^ thymus.

## Discussion

Although the existence of B cells in the thymus has been known for some time, their functional significance has only recently been investigated [34,41,47–49] and the mechanisms controlling their development are not known. As thymic B cells have been reported to play a role in thymic negative selection [33–35], understanding these mechanisms is important to understanding thymus function. Our data from heterochronic transplants show that HSCs from the perinatal period generate higher frequencies and numbers of thymic B cells compared to adults even in the wild-type thymus, indicating that there are stage-specific differences in B cell propensity in HSCs in the bone marrow. In addition, thymi in which *Foxn1* levels decline prematurely have increased numbers of primarily progenitor B cells, indicating that signals from the thymic microenvironment also contribute to specification, proliferation, and differentiation of thymic B cells. This increase is due to an amplification of the underlying B cell competence of the HSCs, and likely due to a combination of signals from TEC that influence B cell development, including decreased Notch signals and increased IL-7. In a related study, we further showed that the timing of these events is also regulated by delayed *Let-7g* up-regulation in LPCs in the thymus, which normally limits the generation of thymic B cells (see *co-submitted manuscript*: *Thymic epithelial cell-derived signals control B progenitor formation and proliferation in the thymus by regulating Let-7 and Arid3a)*, and that this up-regulation also requires FOXN1-dependent signals from the thymic epithelium.

A key event that can influence the differences in thymocyte development between neonatal and adult thymus is the progenitor switch from fetal liver-derived to BM-derived HSCs, which occurs during the neonatal to young adult period [10,11,18]. However, it is as yet unclear when BM-derived adult-type HSCs first seed into the thymus, and how this switch to adult HSCs contributes to thymocyte development during the neonatal period. Both the switch of HSCs from a fetal to adult progenitor profile and maturation of the thymic vasculature had been reported to occur by day 7 postnatal [11,50]. Thus, day 7 is a critical time point for changes to occur in both hematopoietic derived cells and thymus structure that might fundamentally affect thymocyte development during the neonatal period. Consistent with this timing, we observed a wave of DN1a/b cells appearing in the thymus at postnatal day 7 (Fig 2C). Based on current estimates that thymocytes spend 14 days at the DN stages and 2–4 days at the DP stage [51,52], this importation wave of DN1a/b cells would generate a wave of DP thymocytes at around 4 weeks of age. As the importation of BM derived progenitors in the postnatal thymus is a gated phenomenon profiled as 7 days of receptivity and around 4 weeks of refractivity [4,6], and the neonatal BM progenitors posses a high B lineage potential [7,17,18], our data suggest that a wave of neonatal BM progenitors with higher B cell potential enters the thymus during the 1^st^ week after birth to generate a thymic progenitor wave. In a normal microenvironment, these cells generate primarily T cells in thymus, with DP cell production peaking at about day 28, but also a slightly higher incidence of B cells with the same timing. However, in *Foxn1*^*lacZ*^ mutant thymus, due to the defect of thymic microenvironment, this wave of thymic progenitors generated less primarily T cells but increase thymic B cells production peaking at about day 28. After that time, the B cell potential decreases as the wave of progenitors is replaced by the adult-type HSCs in both normal and mutant adult thymus, with lower intrinsic B cell. Our data also support the conclusion that thymic B cells can originate and develop in the thymus, especially during young ages.

Down-regulation of *Foxn1* expression is associated with reduced expression of *MHCII*, *Dll4*, and *Ccl25* in TECs [27,28], all of which we also see in the *Foxn1*^*lacZ*^ mutants. These changes are also consistent with the reduction in MHCII^hi^ TECs [36], which have been previously shown to produce *Ccl25*, associated with progenitor migration to the thymus [53]. The observed reduction of DN1a/b cells in these mutants could thus be directly related to down-regulation of *Ccl25* expression. The gradual increase of thymic B cells in the DN1 population from day 7 to an adult could also be promoted by reduced *Dll4* expression, which is required for T lineage commitment [23,24], one week after birth. However, these changes cannot explain the transient nature of the increase in thymic B cells, or the fact that an increase was seen in both control and *Foxn1*^*lacZ*^ thymus (although the change in controls was slight). The timing of this increase correlated with changes in the B cell potential of BM-derived cells, most clearly demonstrated by the fact that young BM or LSK cells (d 14) showed a higher B lineage potential than adult BM cells when transferred into the B cell permissive *Foxn1* mutant thymic microenvironment. These results are consistent with reports showing that ETPs (DN1a/b cells) derived from neonatal thymus and progenitors from fetal and neonatal BM or cord blood cells possess a high B lineage potential compared to those from adults [21,22,54,55].

The increased numbers and proliferation of these progenitor B cells could be further promoted by the microenvironment in the form of increased *Il7* levels. Exposure to exogenous *Il7* or enforced *Il7* expression has been shown to promote progenitor of B cell development *in* the thymus [32,56,57], and overexpression inhibits the development of TCRαβ T cells [32]. The increase in *Il7* expression seen in our mutants is less obviously due to the down-regulation of *Foxn1* [36], but could be a secondary phenotype, as depletion of thymocytes alone has also been shown to cause an up-regulation of *Il7* and *Cxcl12 (Sdf1a)* in TECs [44,58]. However, reduction of thymocyte number alone is not sufficient to promote thymic B cell production [30]. This would also not account for the dramatic spike in expression at 28 days, which does not correlate with any specific change in either *Foxn1* expression or thymocyte numbers [36]. However, as IL-7 is produced by a specific subset of TECs [44–46,59], and our data show that MHCII^lo^ TECs expressed a higher level of *Il7* in *Foxn1*^*lacZ*^ mutant thymus, it is possible that as the microenvironment declines a subset of TECs are transiently over-represented that produce a high level of IL-7 during neonatal to young adult. Alternatively, it is possible that IL-7 production by TEC is differentially regulated in some way by cross-talk with B cells themselves, and that the spike in IL-7 production is related to the peak of B cell production from fetal HSCs, causing a feed-forward loop. In any case, this high level of IL-7 likely promotes progenitor B cell expansion in the deficient *Foxn1*^*lacZ*^ thymic environment, as these increased thymic B cells were primarily pre-B-II Fr C-D stage [38,40,57,60].

## Conclusion

Taken together, we have shown that neonatal BM progenitors possess a higher B lineage potential than adult type progenitors, that generates a transient wave of B cell development in the thymus during the neonatal to young adult period. This HSC potential was revealed by the thymic microenvironmental deficiency in the *Foxn1*^*lacZ*^ mutants with a reduction of Notch signals and an increase of IL-7 production during this period. Our findings have potential clinical implications for improving the transplantation of umbilical cord blood cells or adult BM cells, and for understanding the contributions of thymic microenvironmental signals to the B cells development and function in the thymus.

## Supporting information

S1 Fig Flow cytometry analysis of thymic B cells after BM cells transfer. **(A-B).** 3000 LSK cells sorted from day 14 BM of CD45.1 mice were retro-orbital transferred into the sub-irradiated 42-day Z/N mice (A), similarly, 3-month LSKs were transferred into 17-day Z/N mice (B) respectively. The profile of CD19 and CD24 staining were showed in donor and host. Data are representative of two independent experiments, (Z/N: n = 3 for A, n = 4 for B). Data are representative of two independent experiments. Student’s t-test results: **P <0.01, ****P <0.0001. Bars indicate means ± SEM.
(TIF)

S2 Fig The cell sorting of BM pre-B cells from BM and thymus. **(A-B).** The total BM cells (A) and total thymocytes (B) from CD45.1 were stained by B220, CD19, CD24, CD43 and IgM + Lin, and the progenitor B cells were sorted on B220^+^CD19^+^ CD24^+^CD43^+/lo^IgM^-^Lin^-^ subpopulation by MoFlo^TM^ cell sorter.
(TIF)

S1 File NC3Rs ARRIVE guidelines checklist. (PDF)
