# Supplementary figures and images for: Thymic B cell development is controlled by the B potential of progenitors via both hematopoietic-intrinsic and thymic microenvironment-intrinsic regulatory mechanisms
Source: PLoS One. 2018 Feb 20;13(2):e0193189. doi: 10.1371/journal.pone.0193189 (PMC5819817; doi:10.1371/journal.pone.0193189)

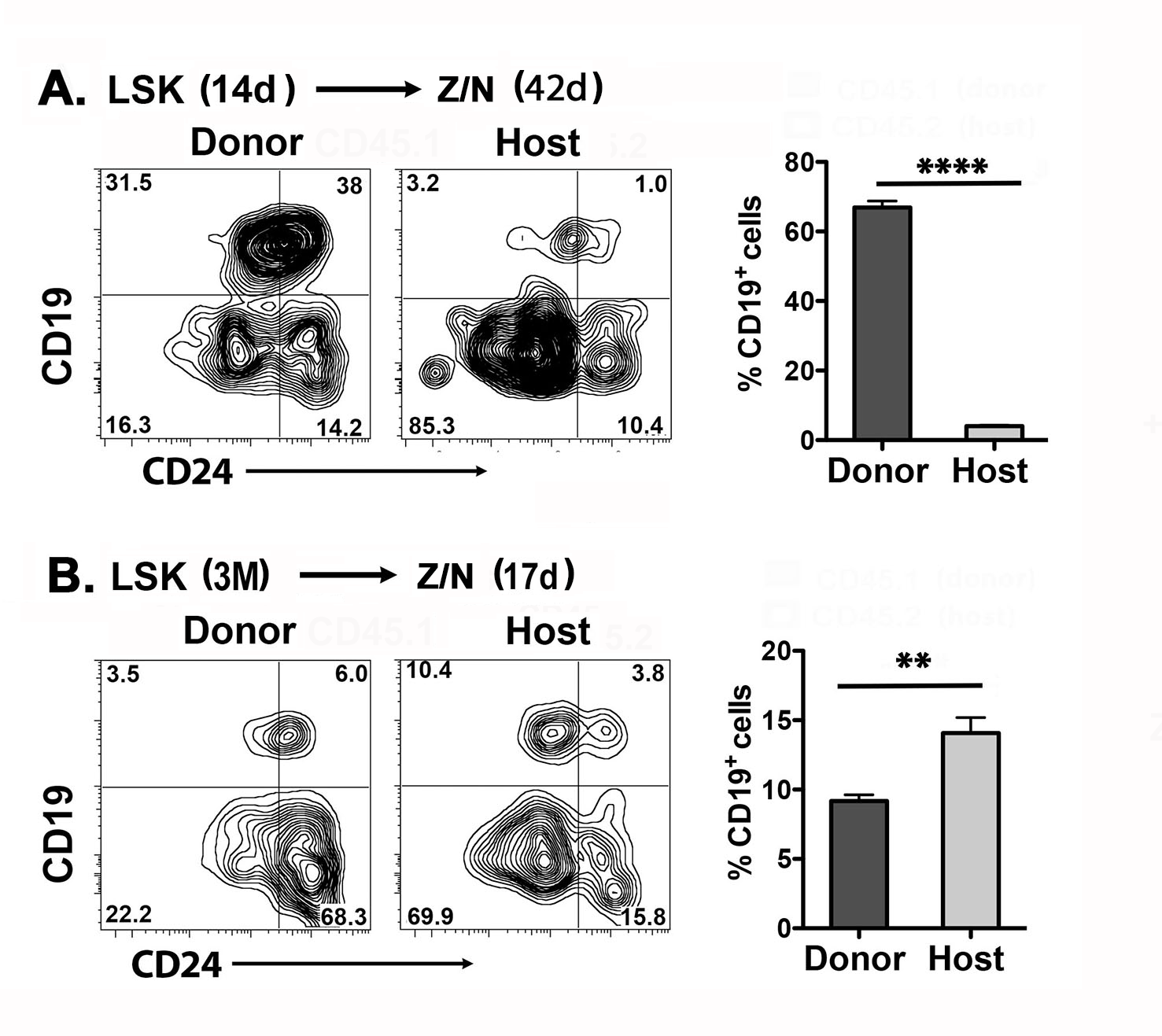

Supplement: S1 Fig — (A-B). 3000 LSK cells sorted from day 14 BM of CD45.1 mice were retro-orbital transferred into the sub-irradiated 42-day Z/N mice (A), similarly, 3-month LSKs were transferred into 17-day Z/N mice (B) respectively. The profile of CD19 and CD24 staining were showed in donor and host. Data are representative of two independent experiments, (Z/N: n = 3 for A, n = 4 for B). Data are representative of two independent experiments. Student’s t-test results: **P <0.01, ****P <0.0001. Bars indicate means ± SEM. (TIF) [file pone.0193189.s001.tif]

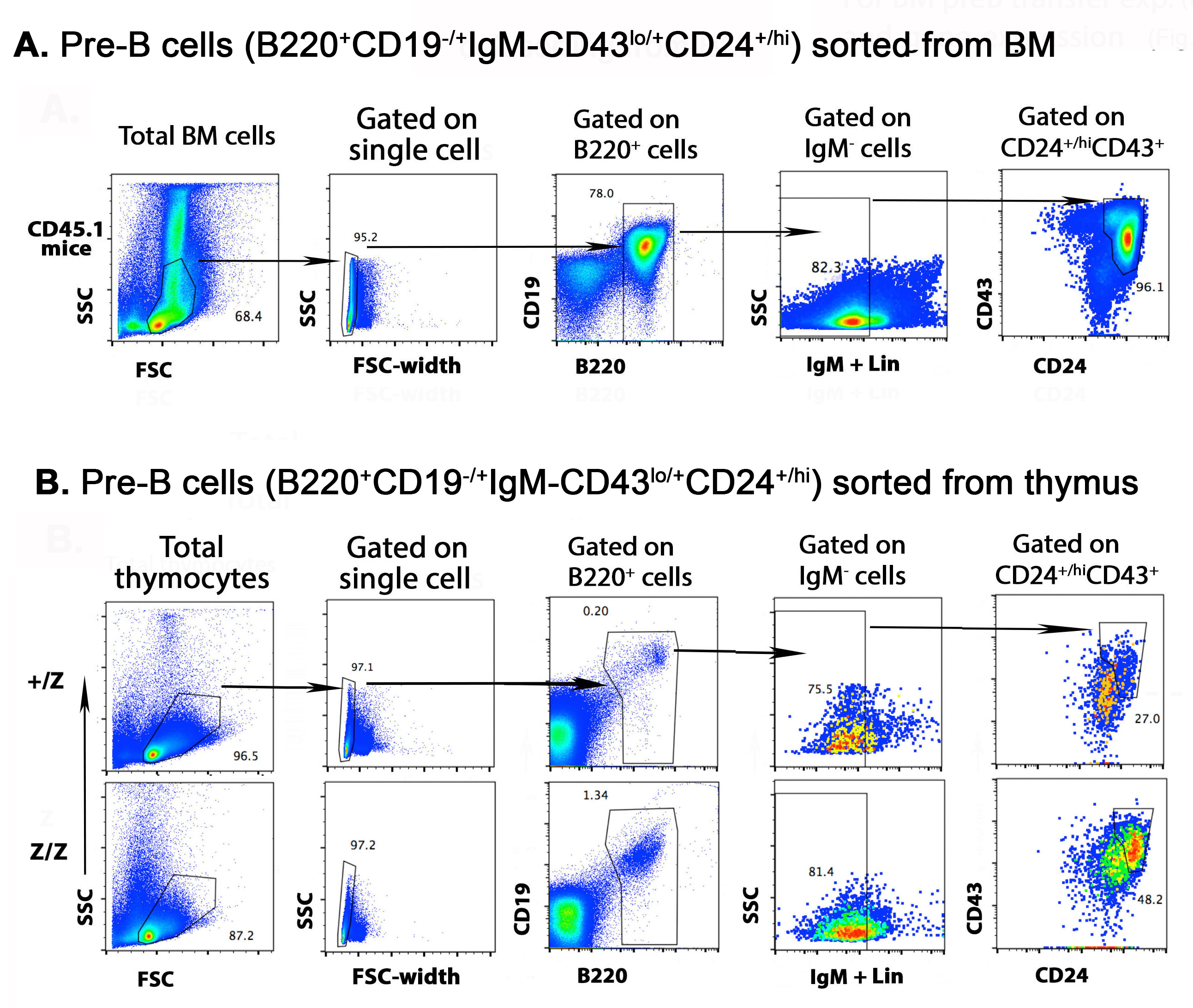

Supplement: S2 Fig — (A-B). The total BM cells (A) and total thymocytes (B) from CD45.1 were stained by B220, CD19, CD24, CD43 and IgM + Lin, and the progenitor B cells were sorted on B220+CD19+ CD24+CD43+/loIgM-Lin- subpopulation by MoFloTM cell sorter. (TIF) [file pone.0193189.s002.tif]
